# Frequency-Following Responses to Speech Sounds Are Highly Conserved across Species and Contain Cortical Contributions

**DOI:** 10.1523/ENEURO.0451-21.2021

**Published:** 2021-12-23

**Authors:** G. Nike Gnanateja, Kyle Rupp, Fernando Llanos, Madison Remick, Marianny Pernia, Srivatsun Sadagopan, Tobias Teichert, Taylor J. Abel, Bharath Chandrasekaran

**Affiliations:** 1 Department of Communication Sciences and Disorders, University of Pittsburgh, Pittsburgh, Pennsylvania 15260; 2 Department of Neurological Surgery, UPMC Children’s Hospital of Pittsburgh, Pittsburgh, Pennsylvania 15213; 3 Department of Linguistics, The University of Texas at Austin, Austin, Texas 78712; 4 Center for Neuroscience, University of Pittsburgh, Pittsburgh, Pennsylvania 15261; 5 Department of Bioengineering, University of Pittsburgh, Pittsburgh, Pennsylvania 15260; 6 Department of Psychiatry, University of Pittsburgh, Pittsburgh, Pennsylvania 15213; 7 Department of Neurobiology, University of Pittsburgh, Pittsburgh, Pennsylvania 15260; 8 Center for the Neural Basis of Cognition, University of Pittsburgh, Pittsburgh, Pennsylvania 15261

**Keywords:** auditory brainstem, auditory cortex, cross-species, frequency following responses, periodicity, pitch

## Abstract

Time-varying pitch is a vital cue for human speech perception. Neural processing of time-varying pitch has been extensively assayed using scalp-recorded frequency-following responses (FFRs), an electrophysiological signal thought to reflect integrated phase-locked neural ensemble activity from subcortical auditory areas. Emerging evidence increasingly points to a putative contribution of auditory cortical ensembles to the scalp-recorded FFRs. However, the properties of cortical FFRs and precise characterization of laminar sources are still unclear. Here we used direct human intracortical recordings as well as extracranial and intracranial recordings from macaques and guinea pigs to characterize the properties of cortical sources of FFRs to time-varying pitch patterns. We found robust FFRs in the auditory cortex across all species. We leveraged representational similarity analysis as a translational bridge to characterize similarities between the human and animal models. Laminar recordings in animal models showed FFRs emerging primarily from the thalamorecipient layers of the auditory cortex. FFRs arising from these cortical sources significantly contributed to the scalp-recorded FFRs via volume conduction. Our research paves the way for a wide array of studies to investigate the role of cortical FFRs in auditory perception and plasticity.

## Significance Statement

Frequency-following responses (FFRs) to speech are scalp-recorded neural signals that inform the fidelity of sound encoding in the auditory system. FFRs, long believed to arise from brainstem and midbrain, have shaped our understanding of subcortical auditory processing and plasticity. Non-invasive studies have shown cortical contributions to the FFRs; however, this is still actively debated. Here we used direct cortical recordings to trace the cortical contribution to the FFRs and characterize the properties of these cortical FFRs. With extracranial and intracranial recordings within the same subjects, we show that cortical FFRs indeed contribute to the scalp-recorded FFRs, and their response properties differ from the subcortical FFRs. The findings provide strong evidence to revisit and reframe the FFR-driven theories and models of subcortical auditory processing and plasticity with careful characterization of cortical and subcortical components in the scalp-recorded FFRs.

## Introduction

Time-varying pitch patterns are a vital component of all spoken languages. Periodicity, a critical cue for time-varying pitch (Plack et al., 2014) can be non-invasively assayed using the scalp-recorded frequency-following responses (FFRs) in animals (Marsh et al., 1975; Smith et al., 1975; Ayala et al., 2017; Teichert et al., 2021) and humans (Coffey et al., 2019). Scalp-recorded FFRs reflect phase-locked activity from neural ensembles along the ascending auditory pathway (Worden and Marsh, 1968; Gerken et al., 1975; Gardi et al., 1979) and provide an integrative and non-invasive snapshot of pitch encoding in neurotypical and clinical populations (Chandrasekaran and Kraus, 2010; Krizman and Kraus, 2019). In neurotypical populations, FFRs have been leveraged to demonstrate experience-dependent shaping of pitch patterns at preattentive stages of auditory processing. Periodicity encoding, as indexed by the FFRs, is found to be atypical in neurodevelopmental disorders (Abrams and Kraus, 2005; White-Schwoch et al., 2015), acquired neurologic disorders (Kraus et al., 2016; Vander Werff and Rieger, 2017), and aging-related decline in auditory processing (Anderson et al., 2012; Bidelman et al., 2014; Presacco et al., 2016; Maruthy et al., 2017). Despite these critical contributions to our understanding of human auditory plasticity and the potential as an easy-to-record non-invasive biomarker, the neural sources of the scalp-recorded FFRs to pitch patterns are poorly understood.

For more than three decades, the inferior colliculus (IC) and the cochlear nucleus were considered the primary neural sources of the FFRs (Marsh et al., 1974; Smith et al., 1975; Yamada et al., 1980; Galbraith et al., 2000; Chandrasekaran and Kraus, 2010; Gnanateja et al., 2012). Brainstem sources of scalp-recorded FFRs have been confirmed using cryogenic cooling of the IC (Smith et al., 1975) and resection of colliculocortical pathways (Greenberg et al., 1981) in animal models. Multichannel electroencephalography (EEG) recordings in humans also show that the subcortical sources predominantly contribute to the scalp-recorded FFRs (Galbraith et al., 2001; Bidelman, 2015, 2018; King et al., 2016). Furthermore, the periodicity code of pitch is thought to be transformed into a rate or rate-place code in the upper brainstem based on FFR studies (Plack et al., 2014). Recent studies using magnetoencephalography (MEG) and EEG have challenged these accounts and shown substantial cortical contributions to the scalp FFRs, with a distinct rightward cortical asymmetry (Coffey et al., 2016, 2021; Hartmann and Weisz, 2019; Gorina-Careta et al., 2021). However, non-invasive MEG and EEG studies require inferences based on distributed source-modeling approaches that are relatively less sensitive to deep brain sources. FFRs to speech stimuli have also been demonstrated in the auditory cortex using direct intracortical recordings (Behroozmand et al., 2016; Guo et al., 2021). While converging evidence shows that FFRs can be recorded from the auditory cortex, it is not known whether these FFRs are generated at the auditory cortex or whether these are volume-conducted electrical fields from the brainstem centers. The existing studies do not inform about the detailed arrangement of the current sources and sinks localized in the auditory cortex that can potentially give rise to these cortical FFRs (cFFRs). Additionally, it remains unclear how far the cortical FFRs are volume conducted and contribute to the scalp-recorded FFRs.

Precise characterization of the laminar sources of FFR is challenging in human participants. Animal models that share anatomic and physiological similarities with the human auditory pathway are invaluable in obtaining fine-grained information about the precise sources of the FFRs. In addition, animal models can also provide the freedom to record intracortical and scalp FFRs in the same animal to further deconstruct the cortical contribution to the scalp FFRs. The rhesus macaque and guinea pig are vocally communicating animals and have been successfully used as animal models to augment our understanding of the FFRs (Yamada et al., 1980; Chou et al., 2014; He et al., 2014; Ayala et al., 2017; Teichert et al., 2021). These animal models are highly similar to humans with respect to audible frequency range, auditory perceptual characteristics, and neuroanatomy (Sinnott et al., 1976; Sinnott and Kreiter, 1991; Kaas and Hackett, 2000; Rauschecker and Tian, 2000; Heffner and Heffner, 2007; Grimsley et al., 2012; Naert et al., 2019). Prior studies using these models have been restricted to the study of subcortical sources of FFRs (Yamada et al., 1980; He et al., 2014; Ayala et al., 2017). Considering the wide applicability of these animal models in understanding human auditory processing, we sought to track the cortical sources of FFRs in the two animal models and examine the extent to which they can aid in understanding FFRs in humans. This further aids in establishing a unified framework for studying the FFR properties, and in leveraging advanced species-specific scientific approaches that can provide different insights into the FFRs in humans.

We used an integrative cross-species [human, rhesus macaque (*Macaca mulatta* [Maq]), and guinea pig [GP]) and cross-level (intracranial and extracranial) approach to deconstruct the cortical contribution to the FFR with unprecedented mechanistic detail. We examined human intracranial recordings with dynamic pitch-varying stimuli (Mandarin tone stimuli) in two participants and confirmed the existence of cortical frequency following responses primarily localized to Heschl’s gyrus (HG). We then applied representational similarity analysis (RSA) to demonstrate striking similarities in FFRs across species (Kriegeskorte et al., 2008; Barron et al., 2021), thereby establishing homologies across animal models to serve as a translational bridge. We further examined FFRs in the two animal models using fine spatial resolution to (1) deconstruct the laminar profile of the cortical sinks and sources of FFRs and (2) quantify cortical contribution to scalp FFRs using intracranial and extracranial recordings in the same animal using blind source separation and spectral profile estimates. Thus, by characterizing the FFRs with such unprecedented mechanistic detail using a cross-species approach, we demonstrated the existence of cortical sources of FFRs that emerge in thalamorecipient layers and contribute to the scalp-recorded FFRs.

## Materials and Methods

### Stimuli: FFRs to Mandarin tones

The syllable /yi/ with four different pitch patterns (tones) were used to elicit the FFRs. These pitch patterns are linguistically relevant in Mandarin and have been extensively used to examine experience-dependent auditory plasticity (Krishnan et al., 2010a, 2012; Lau et al., 2017, 2019; Llanos et al., 2017; Reetzke et al., 2018). The minimally contrastive F0 patterns are phonetically described as T1 (high-level, F0 = 129 Hz), T2 (low-rising, F0 ranging from 109 to 133 Hz), T3 (low-dipping, F0 ranging from 89 to 111 Hz), and T4 (high-falling, F0 ranging from 140 to 92 Hz). These stimuli were synthesized based on the F0 patterns (tones) derived from natural male speech production. All stimuli had a sampling rate of 48,000 Hz and were 250 ms in duration. Stimuli were delivered using ER-3C insert earphones with the volume adjusted to a comfortable intensity level. The stimuli were presented in both condensation and rarefaction polarities to minimize potential contamination of the neural responses by the stimulus artifact and preneural cochlear microphonics (Skoe and Kraus, 2010). A pseudorandom presentation was used where each stimulus had a one-quarter probability of occurrence.

The overall number of stimuli sweeps per tone (T1–T4) differed across species, with at least 250 sweeps obtained in each species (Table 1).

**Table 1:** Details of participants, stimuli, and recording parameters

Parameters	Human sEEG	Human EEG	Macaque intracranial	Macaque extracranial	Guinea pig intracranial	Guinea pig extracranial
Number of participants	2	20	2	2	2	2
Electrode location	Intracranial electrode with cylindrical contacts. Hum1: 129 electrode contacts in bilateral temporal lobes Hum2: 229 electrode contacts in the right hemisphere; temporal, frontal, and parietal lobes	Surface electrodes on the vertex (Cz) and mastoids	Maq1: 96 sharp electrodes on the surface of the auditory and motor cortices Maq2 24 laminar probe electrodes the layers of the auditor cortex	Maq2: 33 EEG electrodes	Laminar probe in the auditory cortex	Surface electrodes at Cz and T4
Transducer	ER-3C	ER-3C	Sound-field speaker	Sound-field speaker	Sound-field Speaker	Sound-field Speaker
Intensity	Comfortable level	75 dB SPL	78 dB SPL	78 dB SPL	75 dB SPL	75 dB SPL
Number of sweeps	800	1000	1000	1000	250	2000 in GP1 250 in GP2
Interstimulus interval	60 ms	128–168 ms	250 ms	250 ms	250 ms	100 ms

### Intracranial and scalp electroencephalography in humans

#### Participants

##### Human participants for sEEG

FFRs were recorded intracranially in Hum1, a 9-year-old boy with drug-resistant epilepsy. The participant was right handed, a native speaker of English, attending grade 4 in school. The participant underwent stereo EEG (sEEG) monitoring of the bilateral temporal lobes for localization of his seizure focus. An opportunity to record sEEG from both temporal lobes to study bilateral auditory processing in the same participant is unique. This participant had no other relevant medical history. To assess generalizability, we also recorded intracranial FFRs in a second participant, Hum2, a 16-year-old boy with drug-resistant epilepsy. The participant was right handed, was a native speaker of English, and had completed grade 9 in school. The participant underwent sEEG monitoring of broad right frontotemporal regions for localization of his seizure foci. In both participants, the choice of electrode insertion was based purely on clinical necessity for evaluation of focal epilepsy. The families of both participants gave written informed consent to participate in the study. All research protocols were approved by the Institutional Review Board of the University of Pittsburgh.

##### Human participants for EEG

Data from a previously published study (Reetzke et al., 2018) with 20 participants in the age range of 18–24 years (12 females) was reanalyzed in this study. All the participants were monolingual native speakers of English. All the participants had hearing sensitivity within 20 dB hearing level across octave frequencies from 250 to 8000 Hz. Written informed consent was obtained from the participants before inclusion in the study. The research protocols used were approved by the Institutional Review Board of the University of Texas, Austin.

#### Electrophysiological recordings in humans

##### Stereoelectroencephalography in humans

sEEG electrodes were inserted into the brain using robot-assisted implantation (Abel et al., 2018; Faraji et al., 2020). Twenty electrode trajectories in Hum1 (129 active electrode contacts) and 18 trajectories in Hum2 (226 active electrode contacts) were inserted along different brain regions to test seizure localization hypotheses based on non-invasive evaluations (Chabardes et al., 2018). Each electrode had between 8 and 12 cylindrical contacts with a length of 2 mm and a diameter of 0.8 mm. The distance between each electrode contact was 3.5 mm. The choice of electrode sampling (spatial resolution) across the trajectories was made based on clinical necessity. Anatomical locations of the electrode sites were obtained using high-resolution computed tomography (CT) and structural MRI. The electrode locations from the CT scan were coregistered with the structural MRI to precisely locate the anatomic locations of each electrode. A cortical reconstruction was generated from the MRI using Freesurfer (Fischl, 2012), and electrodes were localized using CT coregistration in Brainstorm (Tadel et al., 2011). The sEEG signals were recorded with a Grapevine Nomad processor (Ripple) and the accompanying Trellis recording software. The sEEG was recorded at a sampling rate of 1000 Hz, and an online notch filter was applied at 60/120/180 Hz to reduce electrical line interferences. The audio signal was synchronously recorded by the Grapevine system at a sampling rate of 30,000 Hz. The auxiliary audio channel was used to mark the onset times of each stimulus in the sEEG recordings. The participants passively listened to the Mandarin vowels. Neither participant had seizure foci in the temporal lobe or had any active seizure activity during the experiment.

##### Scalp electroencephalography in humans

To understand how the FFRs recorded at the cortex compare with the FFRs that are conventionally obtained from the scalp, we used a scalp-recorded FFR dataset from a previously published study. This dataset also was used to establish a translation bridge between scalp-recorded FFRs and cortical FFRs across humans and animal models. The details of scalp EEG are provided briefly here, and complete details are available in the source article (Reetzke et al., 2018). Scalp-recorded FFRs were recorded from the 20 human participants (10 female) using EEG. EEG was recorded with a single AgCl electrode placed on the scalp that was referenced to the left mastoid, and the ground was placed on the opposite mastoid. The Brainvision EEG system was used to record the EEG activity. A dedicated preamplifier (EP-preamp) connected to the actichamp amplifier with a gain setting of 50×.

### Intracranial and scalp electroencephalography in rhesus macaque

#### Subjects

The EEG experiments and intracortical recordings were performed on two adult male macaque monkeys (Maq1, Maq2). The treatment of the monkeys was in accordance with the guidelines set by the US Department of Health and Human Services (National Institutes of Health) for the care and use of laboratory animals. All methods were approved by the Institutional Animal Care and Use Committee at the University of Pittsburgh. The animals were between 5 and 11 years old and weighed between 8 and 11 kg at the time of the experiments.

#### Cranial EEG recordings

Details of the cranial EEG recordings have been reported previously (Teichert, 2016; Teichert et al., 2016). Briefly, EEG electrodes manufactured in-house from medical grade stainless steel were implanted in 1 mm deep, nonpenetrating holes in the cranium of Maq1. All electrodes were connected to a 36-channel Omnetics connector embedded in dental acrylic at the back of the skull. The 33 electrodes formed regularly spaced grids covering approximately the same anatomy covered by the international 10–20 system (Li and Teichert, 2020). All the electrodes were referenced to an electrode placed at Oz.

#### Intracranial recordings in primary auditory cortex

For the single-tipped sharp electrode recordings in Maq1, neural activity was recorded with a chronically implanted 96-channel electrode array with individually movable electrodes (SC96, Graymatter). For the laminar recording in Maq2, neural activity was recorded with a 24-channel laminar electrode (S-Probe, Plexon) positioned approximately perpendicular to the orientation of the left superior temporal plane. The depth of the probe was adjusted iteratively until the prominent sound-evoked supragranular source was located slightly above the center of the probe. At the time of the experiments, 12 of the electrodes were positioned in or close enough to the superficial layers of the auditory cortex to pick up frequency-tuned local field potentials (LFPs). Six of these electrodes also picked up frequency-tuned multiunit activity (MUA), suggesting that they were located in layer III or below. The devices in both animals were implanted over the right hemisphere in a way that allowed electrodes to approach the superior temporal plane approximately perpendicular.

#### Experimental setup

All experiments were performed in small (4 feet wide × 4 feet deep × 8 feet high) sound-attenuating and electrically insulated recording booths (Eckel Noise Control Technologies). Animals were positioned and head-fixed in custom-made primate chairs (Scientific Design). Neural signals were recorded with a 256-channel digital amplifier system (model RHD2000, Intan) at a sampling rate of 30 kHz.

Experimental control was handled by a Windows PC running an in-house modified version of the MATLAB software package monkeylogic. Sound files were generated before the experiments and presented by a subroutine of the MATLAB package Psychtoolbox. The sound files were presented using the right audio channel of a high-definition stereo PCI sound card (model M-192, M-Audiophile) operating at a sampling rate of 96 kHz and 24 bit resolution. The analog audio signal was then amplified by a 300 W amplifier (QSC GX3). The amplified electric signals were converted to sound waves using a single-element 4 inch full-range driver speaker (model W4-1879, Tang Band) located 8 inches in front of the animal and presented at an intensity of 78 dB SPL. To determine sound onset with high accuracy, a trigger signal was routed through the unused left audio channel of the sound card directly to one of the analog inputs of the recording system. The trigger pulse was stored in the same stereo sound file and was presented using the same function call. Hence, any delay in the presentation of the tone also leads to an identical delay in the presentation of the trigger. Thus, sound onset could be determined at a level of accuracy that was limited only by the sampling frequency of the recording device (30 kHz, corresponding to 33 μs).

### Cranial electroencephalography and intracranial recordings in guinea pigs

#### Subjects

The cranial EEG and intracranial recordings were performed on two wild-type (age ∼8 months), pigmented GPs (GP1 and Gp2; Cavia porcellus, Elm Hill Labs), weighing ∼600–800 g. All experimental procedures were conducted according to National Institutes of Health *Guidelines for the Care and Use of Laboratory Animals* and were approved by the Institutional Animal Care and Use Committee of the University of Pittsburgh.

Before commencing recordings, a custom headpost for head fixation, skull screws that served as EEG recording electrodes, or reference electrodes for intracranial recordings, and recording chambers for intracranial recordings were surgically implanted onto the skull using dental acrylic (C & B Metabond, Parkell) following aseptic techniques under isoflurane anesthesia. Analgesics were provided for 3 d after surgery, and animals were allowed to recover for ∼10 d. Following recovery, animals were gradually adapted to the recording setup and head fixation for increasing durations of time.

#### Experimental setup

All recordings were performed in a sound-attenuated booth (IAC) whose walls were covered with anechoic foam (Pinta Acoustics). Animals were head fixed in a custom acrylic enclosure affixed to a vibration isolation tabletop. Stimuli were presented using MATLAB (MathWorks). Digital stimulus files sampled at 100 kHz were converted to an analog audio signal (National Instruments), attenuated (Tucker-Davis Technologies), power-amplified (Tucker-Davis Technologies), and delivered through a calibrated speaker (4 inch full-range driver, Tang Band) located ∼0.9 m in front of the animal. Stimuli were presented at ∼75 dB SPL.

#### Cranial EEG recordings

FFRs were acquired from unanesthetized, head-fixed, passively listening GPs using a vertical electrode montage. Scalp-recorded activity was collected via a stainless steel skull screw (Fine Science Tools). Reference and ground conductive adhesive hydrogel electrodes (Foam electrodes, Kendall or Covidien Medi-Trace) were placed on the high forehead and mastoid, respectively. Signals were acquired using a multichannel neural processor (Ripple).

#### Intracranial recordings in primary auditory cortex

Intracortical recordings were performed in unanesthetized, head fixed, passively listening GPs. Small craniotomies (diameter, 1–2 mm) were performed within the implanted recording chambers over the expected anatomic location of primary auditory cortex (PAC). Neural activity was recorded using high-density 64-channel multisite electrode (Cambridge NeuroTech), inserted approximately perpendicular to the cortical surface. The tip of the probe was slowly inserted to a depth of ∼2 mm. After a brief waiting period to allow the tissue to settle, signals were acquired using a multichannel neural processor (Ripple Scout).

A summary of the participant information, stimulus, and acquisition parameters across the three species are provided in Table 1.

### Data processing and analyses

#### FFR preprocessing and analysis for sEEG in humans

A linear regression-based method was used to remove the harmonics of the power line interference from the data, using the cleanline plugin in EEGLAB (Delorme and Makeig, 2004). The raw sEEG was high-pass filtered using a third-order zero-phase shift Butterworth filter. No low-pass filter was used, as the sampling rate was 1000 Hz, resulting in an effective low-pass frequency of 500 Hz. Time-locked sEEG epochs were extracted for all vowels in both polarities. The epochs that exceeded amplitudes of 75 μV were rejected. The FFRs in both the polarities for each vowel were averaged to obtain a total of four FFR waveforms (one for each vowel). Four FFR waveforms were obtained for each electrode.

**Figure 1.**
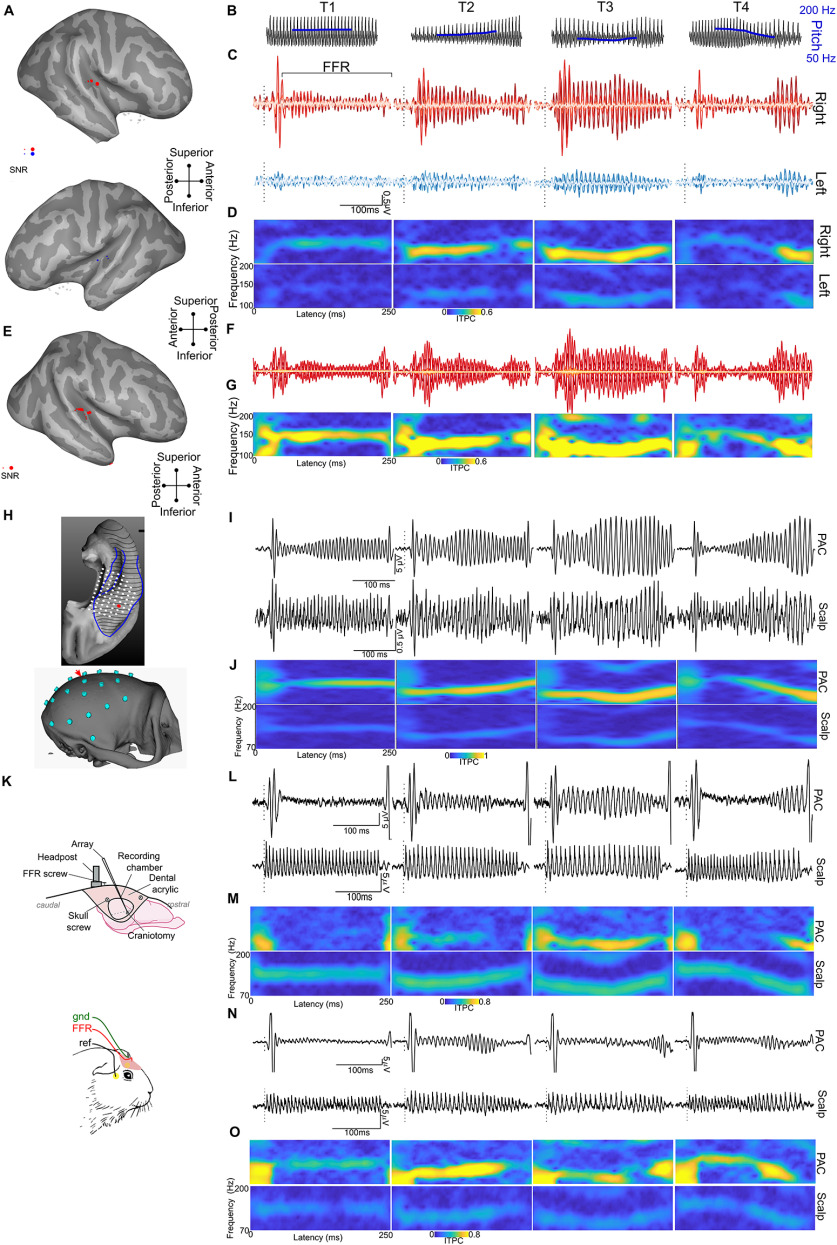
Cross-species and cross-level approach to characterizing the functional properties of cortical FFRs. ***A–O***, Human data (***A*–*G***), macaque data (***H–J***), guinea pig data (***K–O***). ***A*** and ***E*** show the location of the sEEG electrodes (projected to the surface for visualization) projected on the inflated brain surfaces of the human participants Hum1 and Hum2, respectively. Electrodes in gray do not show any significant FFRs above the prestimulus baseline (*p* < 0.01 on paired *t* tests on bootstrapped samples). Electrodes marked in red or blue show significant FFR magnitudes above the prestimulus baseline, where the size of the marker is proportional to the signal-to-noise ratio for FFRs. ***B***, Waveforms of the Mandarin tones/yi/; T1 (high-level F0), T2 (low-rising F0), T3 (low-dipping F0), and T4 (high-falling F0). ***C***, ***F***, FFR waveforms from sEEG electrodes in both right (top: red) and left (bottom; blue) temporal lobes in in Hum1 and Hum2, respectively. ***D***, ***G***, FFR ITPC at all time points and frequencies with a spectrogram for the sEEG FFRs in Hum1 and Hum2, respectively. The ITPC spectrograms shown are only for the electrodes with the highest FFR amplitude in each hemisphere (located on the HG). ***H***, Top, Locations of the semichronic sharp electrodes (white dots) used to record FFRs in the PAC of the macaque. FFRs from a representative example electrode highlighted in red. Bottom, Layout of the EEG Electrode grid. FFR responses analyzed here were from the electrode marked with the red arrow. ***I***, Waveforms show simultaneously recorded FFRs from macaque PAC (top) and skull (bottom, approximately Cz). ***J***, ITPC spectrograms of FFRs in the macaque at both PAC and scalp. ***K***, Intracranial and extracranial electrode setup in the guinea pigs GP1 and GP2. ***L***, ***N***, FFR waveforms to the four Mandarin tones recorded from the best laminar depth electrode placed in the PAC (top) and from a surface scalp electrode (bottom) of the guinea pigs GP1 and GP2, respectively. ***M***, ***O***, ITPC spectrograms of FFRs in guinea pigs GP1 and GP2.

The intertrial phase coherence (ITPC) was estimated to assess the frequencies at which the cortical units phase-lock. The single-trial FFRs were decomposed into a spectrogram representation using time–frequency analysis timefreq.m in EEGLAB. Specifically, 130 wavelets between 70 and 200 Hz with equal widths were used for the time–frequency decomposition. The complex valued time–frequency vectors were divided by their magnitude to obtain unit vectors at every frequency and time point. These unit vectors in the time–frequency domain were averaged across trials to obtain resultant vectors. The absolute magnitude of the resultant vectors at each time and frequency point was used to obtain the ITPC spectrogram (Fig. 1). ITPCs ranged from 0 to 1, with 0 indicating no phase-locking and 1 indicating perfect phase-locking across trials. These ITPCs provide information about the extent of phase-locking at different frequencies and latencies without the confounds of differences in absolute FFR magnitude.

#### FFR processing and analysis for EEG in humans

The EEG data from the humans were bandpass filtered from 80 to 1000 Hz and epoched from −25 to 250 ms (re: stimulus onset). Baseline correction was applied on each epoch, and the epochs exceeding an amplitude of ±35 μV were excluded from further analysis.

#### FFRs to Mandarin tones in macaques and guinea pigs

The raw data were high-pass filtered using a second-order zero-phase shift FIR filter. Time-locked epochs were extracted for all vowels in both polarities. The epochs that exceeded amplitudes of 250 μV were rejected. The FFRs in both the polarities for each tone were averaged to obtain a total of four FFR waveforms (one for each tone).

Custom MATLAB and R routines were used to filter and average signals appropriately to obtain LFPs and MUA from the macaque and GP recordings. The current source density (CSD) in the macaque was computed from the LFP signals derived from the electrodes with 150 μm spacing, spatially smoothing the LFP signal using a Gaussian filter (SD = 250 μm) and obtaining the second spatial derivative method using the finite difference approximation. In the GP, CSD was computed from the LFP signals derived from alternate electrode contacts (60 μm spacing), spatially smoothing the LFP signal using a Gaussian filter (SD = 125 μm) and obtaining the second spatial method using the finite difference approximation. The sink with the earliest latency in the CSD post-stimulus onset was used to identify the thalamorecipient layers.

#### RSA of FFRs to Mandarin tones

RSA was used to establish homologies between the species and assess similarities across scalp and cortical FFRs. RSA was performed on the accuracies of a machine-learning model to decode the four mandarin tones (pitch patterns) based on the FFRs. A hidden Markov model (HMM) classifier was used as the machine-learning model and was trained to decode the Mandarin tones based on the FFR pitch tracks (Llanos et al., 2017). A detailed description of the HMM-based decoding approach can be found in a previously published methods article (Llanos et al., 2017). The averaging size of the HMM was dynamically adjusted to obtain equivalent classification accuracies across the different levels (scalp, cortex) and species. The averaging sizes used were 150 trials for human scalp FFR; 24 and 2 trials for sEEG FFR in Hum1 and Hum2, respectively; 150 trials for macaque scalp FFR; 4 trials for macaque PAC FFR; 6 trials for the guinea pig scalp FFRs; and 16 trials for the GP PAC FFRs. The confusion matrices of decoding patterns (proportion correct) were extracted and used for further representational similarity analysis. Multidimensional scaling (MDS) analysis was performed on the confusion matrices (diagonals removed) to assess whether the decoding patterns across levels and species were similar. Procrustes analysis was performed to rotate and transform all the MDS representation to the same scale to facilitate comparison across species and levels. A similarity matrix was derived from the confusion matrices (diagonals removed) across levels and species using Pearson product-moment correlations, and the significance of these correlations was also assessed.

#### Comparison of FFRs recorded at the scalp and the auditory cortex: spectrotemporal measures

In the animal models, we had the opportunity to record FFRs from the scalp and the cortex in the same animal (Maq1, GP1, and GP2). We compared the FFR power spectra at the scalp and cortex to analyze similarities and differences in the spectral composition between the scalp and cortical FFRs. Welch’s power spectral density (PSD) estimates of the FFRs were obtained with a 1024 point Hamming window with 50% overlap to obtain a smoothed spectral estimate of the FFRs. The PSDs of all four tones were averaged to obtain an average spectral composition of the FFRs. The PSD of the scalp and cortical FFRs were normalized by setting their maximum magnitude to 0 dB. This facilitated the comparison of the spectral decay and relative differences in encoding of the F0 and the higher harmonics at the scalp and the cortex. Because of the high amplitude of the FFR at the F0, the normalization process essentially normalized the F0 magnitude, which facilitated the inspection of decay in the magnitude of the high-frequency components in the FFRs with reference to the F0 magnitude.

Because of time-varying F0 trajectories in the stimuli, it is challenging to infer the FFR temporal properties from a singular estimate of cross-correlation latency based on the raw FFR waveforms. This is especially challenging when contributions of different underlying sources of FFRs are also expected to change depending on stimulus frequency. Thus, we compared the latencies between the scalp and cortical FFRs in the time–frequency domain. We decomposed the FFRs into a time–frequency representation using the continuous wavelet transformation with the Morse wavelet implementation in the MATLAB wavelet analysis toolbox. The real-valued, wavelet-decomposed waveforms at every frequency were then cross-correlated between the scalp and cortical FFRs. The cross-correlation lags with the highest absolute correlation coefficient were estimated. This analysis provided a latency estimate (cross-correlation lag) and magnitude of similarity (Pearson’s *r*) for every frequency in the FFRs. The latencies measured here are with the scalp FFRs as reference. Thus, positive latencies indicate that the cortical FFRs lag scalp FFRs in latency. One scalp and one intracortical electrode with the best signal-to-noise ratio (SNR) were chosen for this analysis. In Maq1, scalp and intracortical FFRs were obtained simultaneously. In GP1, scalp and intracortical FFRs were obtained in two separate sessions, while in GP2 both scalp and intracortical FFRs were obtained simultaneously. The FFRs from the cortex were derived from the electrode with the maximum signal-to-noise ratio in laminar FFR-LFP recordings.

A cross-spectral power density estimate was also obtained to assess the similarity in power between the scalp and cortical FFRs regardless of the latency difference. This estimate is useful in getting an objective metric of similarity in spectral properties of the FFRs at the cortex and the scalp, regardless of the differences in temporal properties. The cross-spectral densities were obtained using Welch’s periodogram method with 50% overlapping 1024-point Hamming windows. The absolute power of the cross-spectral densities was averaged across the FFRs for the four Mandarin tones and overlaid on the plot of frequency-wise latency comparison to obtain a unified inference of the spectrotemporal similarities in the FFRs at the scalp and cortex.

The above measures show differences and similarities in the scalp and cortically recorded FFRs. Multiple volume-conducted fields in the cortex and subcortex that lead to constructive and destructive interferences drive these differences and similarities, and the above measures may not be sensitive to differentiate these fields. Thus, we further used a blind source separation approach using independent component analysis (ICA) that can separate spectrotemporally overlapping components arising from different neural sources (Makeig et al., 2004). ICA extracts mutually independent components disentangling the superposed electrical fields from the different electrical dipoles across the auditory cortical subcortical regions and their projection to the scalp and the cortical electrodes. While the electrodes placed at the cortex record activity from the cortical ensembles in their proximity, they also pick up electrical fields from the subcortical regions and the surrounding auditory cortical fields. With ICA, we can decompose the neural sources underlying the electrical activity across the cortical and scalp electrodes, and trace the differential projection of neural sources to the individual electrodes.

ICA was applied on 33 scalp electrodes and 96 sharp electrodes from the cortical surface (spanning the surface of superior temporal plane and motor cortices) in the macaque and 25 laminar depth electrodes in the auditory cortex and two scalp electrodes (Cz and T4) in the guinea pig. The averaged FFRs corresponding to all four Mandarin tones were input into the ICA decomposition. The Infomax algorithm (runica.m) in EEGLAB toolbox (Delorme and Makeig, 2004) was used for ICA decomposition of the neural data. A principal component analysis was performed to reduce the dimensionality of the signal and to restrict the decomposition to components that explained 96% (macaque) and 99% (guinea pig) of the original variance in the data. The percentage variance accounted for (PVAF) by each ICA was estimated (eeg_pvaf.m). The ICAs that each explained >10% of the variance in the FFRs were retained for further analysis. The spatial weights of the ICAs were derived as the pseudoinverse of the product of the ICA weights and the ICA sphering matrix. These spatial weights were used to assess the spatial layout of the volume-conducted propagation of the different ICAs. The PVAFs of the ICAs at each scalp and cortical electrode were estimated to obtain the contribution of the ICAs to each of the electrodes. The emphasis of this analysis was to assess the percentage contribution of the cortical ICAs to the scalp-recorded FFRs. The latencies of the ICAs were assessed by cross-correlation with the stimulus waveform. These latencies were also used to infer the potential generators of the ICAs, with earlier latencies corresponding to more subcortical sources. The power coherence was estimated between each of these ICAs and the stimulus waveform using cross-power spectral density (cpsd.m). This analysis aided in inferring the differential pattern in the decline of power coherence across the cortical ICAs and subcortical ICAs. The power coherence metric is not a measure of phase-locking but just the power coherence between the stimulus and the ICAs.

### Data availability

The analysis codes will be provided to readers on request.

## Results

### FFRs in the human auditory cortex to vowels with time-varying pitch contours

FFRs were recorded from stereotactically implanted electrodes (Fig. 1*A*,*E*) in two participants while they listened to the pitch-varying Mandarin vowels. In both participants, the location of the electrodes was based on clinical necessity. In Hum1, the electrodes were implanted in both hemispheres with electrodes spanning across the superior temporal plane, superior temporal gyri/sulci, middle temporal gyri/sulci, and insula. In Hum2, the electrodes were implanted only in the right hemisphere spanning the frontal, parietal, and temporal lobes, the superior temporal plane, and insula.

**Figure 2.**
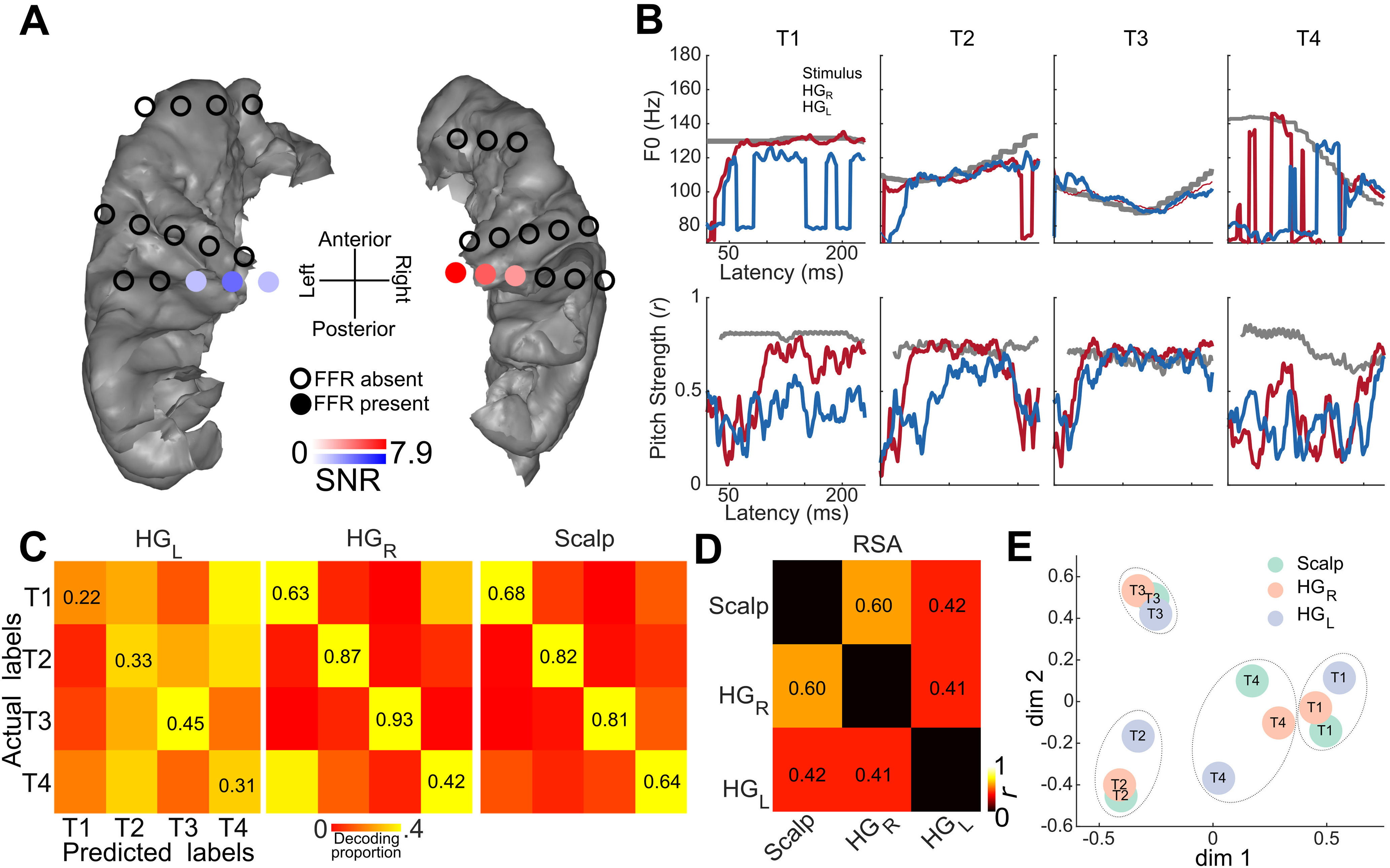
Hemispheric differences in the FFRs in Hum1. ***A***, Electrodes implanted in the superior temporal plane are shown with filled circles marking significant FFR root mean square amplitudes above the prestimulus baseline (*p* < 0.05 permutation-based bootstrapped *t* tests) and unfilled circles mark electrodes where the FFRs were not significantly above the baseline. ***B***, Pitch-tracking measures (sliding window autocorrelation analysis) of the FFRs from the best electrodes in the two hemispheres showing the hemispheric differences. Top row shows the derived F0 track in hertz (based on best lag in each sliding window) in the right HG (HG_R_) and left HG (HG_L_). Bottom row shows the pitch strength (maximum correlation coefficient in each sliding window). ***C***, Hidden Markov model-based confusion matrices showing the decoding accuracies of the Mandarin tone from the FFRs recorded in the right and left Heschl’s gyri from the sEEG recording, and scalp EEG recordings from 20 different participants. ***D***, Representational similarity (Pearson’s correlation) between the FFR confusion matrices (diagonals removed) in ***C***. The correlation between HG_R_ and scalp FFR was significant (*p* = 0.039), while the other correlations were not significant. ***E***, Multidimensional scaling of the confusion matrices in ***C***, visualizing the confusion space for decoding the Mandarin tone.

We analyzed time-locked and phase-locked neural activity to the periodicities in the stimulus. Robust FFRs (Fig. 1*C*,*F*) with amplitudes above prestimulus baseline (*p *<* *0.05 on permutations-based *t* tests between prestimulus baseline and FFRs on bootstrapped FFR trials) were observed in the electrode contacts in the HG and the planum temporale (PT) in both subjects (6 of 129 electrode contacts in Hum1, and 10 of 226 electrode contacts in Hum2; Figs. 1*A*, 2*E*). Electrode contacts farther from HG did not show FFR like responses that were significantly above the prestimulus baseline level (*p *<* *0.05 on permutations-based *t* tests between prestimulus baseline and FFRs on bootstrapped FFR trials). Thus, further FFR analyses were restricted to the electrodes along the HG.

We used four-pitch variants of the vowel/yi/ (Fig. 1*B*), referred to as Mandarin tones, to elicit the FFRs (Fig. 1*C*). These Mandarin tones have been extensively used to record FFRs to study the neurophysiology of pitch processing and associated plasticity in humans (Krishnan et al., 2010b; Llanos et al., 2017; Reetzke et al., 2018; Lau et al., 2019). The Mandarin tones are phonetically described as follows: T1 (high-level F0), T2 (low-rising F0), T3 (low-dipping F0), and T4 (high-falling F0). Morphologically, the time-locked averaged sEEG responses to the Mandarin tones showed robust onset responses followed by FFRs that lasted throughout the stimulus duration. We refer to the FFRs recorded from electrode contacts in close proximity to or directly within the auditory cortex as cFFRs from here on. As is the case for scalp-recorded FFRs, the cFFRs closely followed the fundamental frequency of Mandarin tones (Fig. 1*B*). All four Mandarin tones elicited robust cFFRs (Fig. 1*C*,*F*) in the electrode trajectories that were inserted along the HG, PT, and superior temporal gyrus. The cFFRs that showed the highest amplitudes and signal-to-noise ratios were found in the electrode contacts closest to HG (Fig. 1*A*,*E*; *p *<* *0.05, permutation-based ANOVA followed by *post hoc* paired *t* tests on bootstrapped trials).

The magnitudes of the cFFRs were highest for tones with lower F0 (i.e., most robust in T3; 89–111 Hz) and T2 (109–133 Hz), followed by T1 (∼129 Hz) and T4 (140-92 Hz; Fig. 1*D*). This pattern is clearly visualized within the cFFRs to T2 and T4, where strong ITPC or phase-locking can only be observed when the F0 of the vowel is low and phase-locking declines when the F0 is high (Fig. 1*D*,*G*).

### Cortical FFR latencies in human sEEG do not reflect volume-conducted activity from the brainstem

We assessed the latencies of the cFFRs for vowel T3 as they were the strongest and present throughout the stimulus duration. The latencies of the onset portion of the cFFRs were 14–16 ms, which is much later than the latencies expected of brainstem responses. Similar to the onset latencies, cFFR latencies (based on cross-correlation lags with maximum correlation coefficient) in the right HG (Liégeois-Chauvel et al., 1994) were ∼13–26 ms. These latencies are not consistent with the earlier neural conduction delays expected of inferior colliculus activity and suggest that the recorded cFFRs reflect phase-locking of postsynaptic potentials in cortical neurons.

### Hemispheric asymmetry in cFFRs

Hemispheric asymmetry was analyzed in Hum1 with bilateral temporal lobe coverage. The high-quality and high signal-to-noise ratio FFR data allowed us to statistically assess hemispheric asymmetry within the subject. cFFRs to the Mandarin tones showed a distinct hemispheric asymmetry, consistent with prior studies using MEG (Coffey et al., 2016; Gorina-Careta et al., 2021). The electrodes in the right hemisphere showed higher-amplitude cFFRs to the Mandarin tones (*p *<* *0.01, permutation-based *t* tests on signal-to-noise ratios on bootstrapped cFFR samples). The rightward symmetry was also seen in the ITPC spectrograms and pitch-tracking accuracy to the Mandarin tones (Figs. 1*D*, 2*B*), which together indicate better phase-locking to the stimulus F0 in the right hemisphere. We used an HMM to decode the mandarin tones from the cFFRs. The cFFRs from the right hemisphere tracked the stimulus pitch better than in the left hemisphere (Fig. 2*B*). Consequently, decoding accuracies were higher in the right hemisphere than the left hemisphere (Fig. 2*C*). The pattern of tone decoding errors (“confusions”) correlated significantly (*p* < 0.05) between cFFRs from the right hemisphere and the scalp FFRs from a set of 20 subjects, but the same was not true for the cFFRs from the left hemisphere and the scalp FFRs (Fig. 2*D*). Despite this difference, multidimensional scaling analysis revealed similar clustering of tone FFRs across the scalp, right HG, and left HG (Fig. 2*E*).

### RSA of cross-species and cross-level FFRs

As in the humans, we recorded EEG in both animal model systems to establish scalp-derived FFRs as a translational bridge among the three species. Recordings in the animal models used the same Mandarin tone stimuli previously used for the human subjects. The scalp-recorded FFRs in both model species showed FFR activity (Fig. 1*I*,*L*,*N*) above the prestimulus baseline (SNR_Maq_ = 3.2, SNR_GP1_ = 7, SNR_GP2_ = 3.4) and showed the expected phase-locking to the F0 of the stimuli (Fig. 1*J*,*M*,*O*). Both species showed FFRs that correlated (*r*_Maq_ = 0.45, *r*_GP_ = 0.48, *r*_GP1_ = 0.5, *r*_GP2_ = 0.5; *r* maximum cross-correlation coefficient) with the stimulus at latencies (Lat_Maq_ = 3 ms, Lat_GP_ = 3.5 ms) expected of early brainstem responses.

Furthermore, we recorded LFPs from electrodes in PAC to compare against the intracranial LFP recordings in the human epilepsy patient. Similar to the scalp-recorded FFRs, the intracranial LFPs in both model species also yielded strong amplitude cFFRs above the prestimulus baseline (SNR_Maq_ = 18.3, SNR_GP1_ = 3.7381, SNR_GP2_ = 7.7) and readily showed the expected phase-locking to the F0 of the stimuli (Fig. 1*J*,*M*,*N*). The latencies of the cFFRs (Lat_Maq_ = 11.6 ms, Lat_GP1_ = 9.7 ms, Lat_GP2_ = 10.1 ms), however, were longer than scalp-recorded FFRs in both species (*p* values* *<* *0.001 in both GPs and Maqs; on sign rank comparison of stimulus to response cross-correlation latencies on bootstrapped samples).

**Figure 3.**
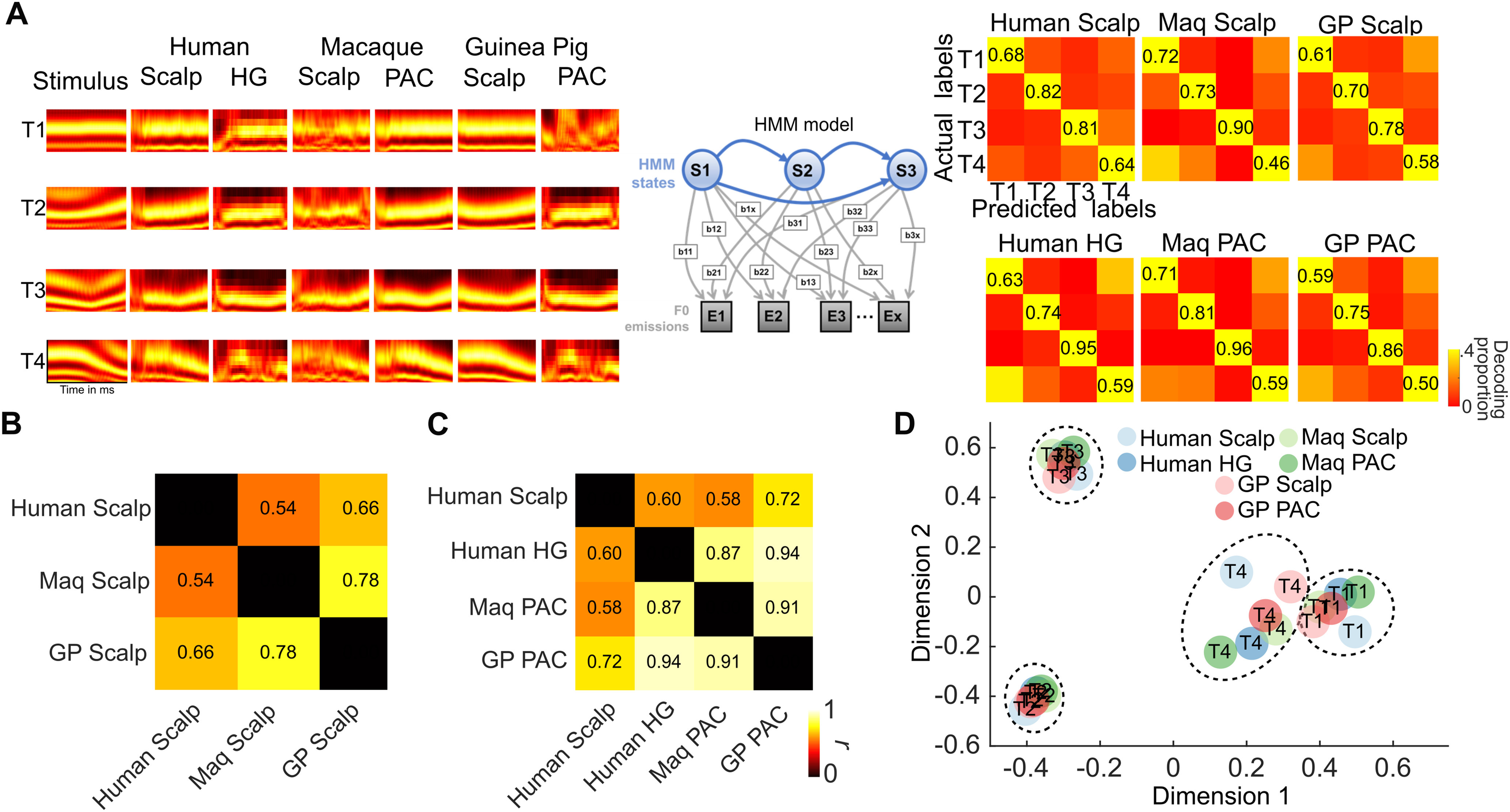
Cross-species and cross-level representational similarity analyses of FFRs. ***A***, An HMM was used to assess the extent to which pitch patterns (T1-high flat, T2-low rising, T3-low dipping, and T4-high falling) could be decoded from the FFRs. HMM decoding accuracies and confusion matrices were estimated for each species (human, macaque, and guinea pig) and each level (scalp and intracortical). HMM decoding accuracies were significantly above chance for all species, levels. Confusion matrices (right) show the accuracies for decoding (along the diagonal) the pitch patterns from FFRs and error patterns. The averaging size used for decoding was adjusted to obtain comparable classification accuracies at the scalp and the cortex. The confusion matrices in ***A*** (principal diagonal removed) were assessed for correlations (Pearson’s *r*) between species and between levels to estimate the cross-species and cross-level representational similarity. ***B***, The correlation between the confusion matrices for scalp FFR across species. FFRs recorded at the scalp share strong similarities (*p* values* *<* *0.05). However, the correlation between human and macaque scalp-recorded FFR did not reach statistical significance (*p* = 0.06). ***C***, The correlation between the confusion matrices of intracortical FFRs across species. Intracortical FFRs showed very strong correlations (*p* values* *<* *0.05). Also shown is the correlation of intracortical FFRs across species and the scalp-recorded FFRs in humans. The human scalp FFRs and GP intracortical FFRs did not show a significant correlation (*p* = 0.049). ***D***, MDS analysis of the pitch patterns based on the confusion matrices in each species and level.

We used RSA to quantify similarities between humans and animal models across different recording levels (intracortical vs scalp; Fig. 3). RSA was performed on confusion matrices constructed from FFRs recorded using harmonized stimuli (four Mandarin tones) across species and levels. Human scalp data were derived from FFRs recorded in 20 participants from a previously published study (Reetzke et al., 2018). In the macaque and GP subjects, scalp FFRs were recorded from cranial EEG electrodes surgically implanted in the skull. In the macaque, intracranial data were recorded from an electrode positioned immediately above layer 1 of the PAC from a chamber implanted over the frontal cortex. In the GP, intracranial data were recorded from an electrode contact estimated to be positioned in putative layer 4 of PAC. Visual inspection shows that the pattern of phase-locking of FFR and cFFR in the animal models was similar to that seen in the human, with phase-locking declining rapidly with increasing stimulus F0 (Fig. 1*D*,*G*,*J*,*M*,*O*).

We decoded the Mandarin tone categories from scalp and intracranially recorded FFRs for all species using an HMM classifier (Llanos et al., 2017; Reetzke et al., 2018). The HMM classifier performed at above-chance levels (>0.25) across species and levels in identifying the correct pitch patterns from the FFRs (Fig. 3*A*, principal diagonal). Human and animal FFR confusion matrices were strikingly similar at both the scalp (Fig. 3*B*) and intracortical levels (Fig. 3*C*; *p* < 0.05, Pearson’s correlation of confusion matrices without the principal diagonal) with stronger similarity seen for the intracortical cFFRs (Fig. 3*C*). However, the scalp FFRs also yielded subtle species-specific differences (with greater similarity between human and GPs, relative to the macaque model).

### High-density intracortical recordings in animal models reveal the laminar distribution of cFFRs

**Figure 4.**
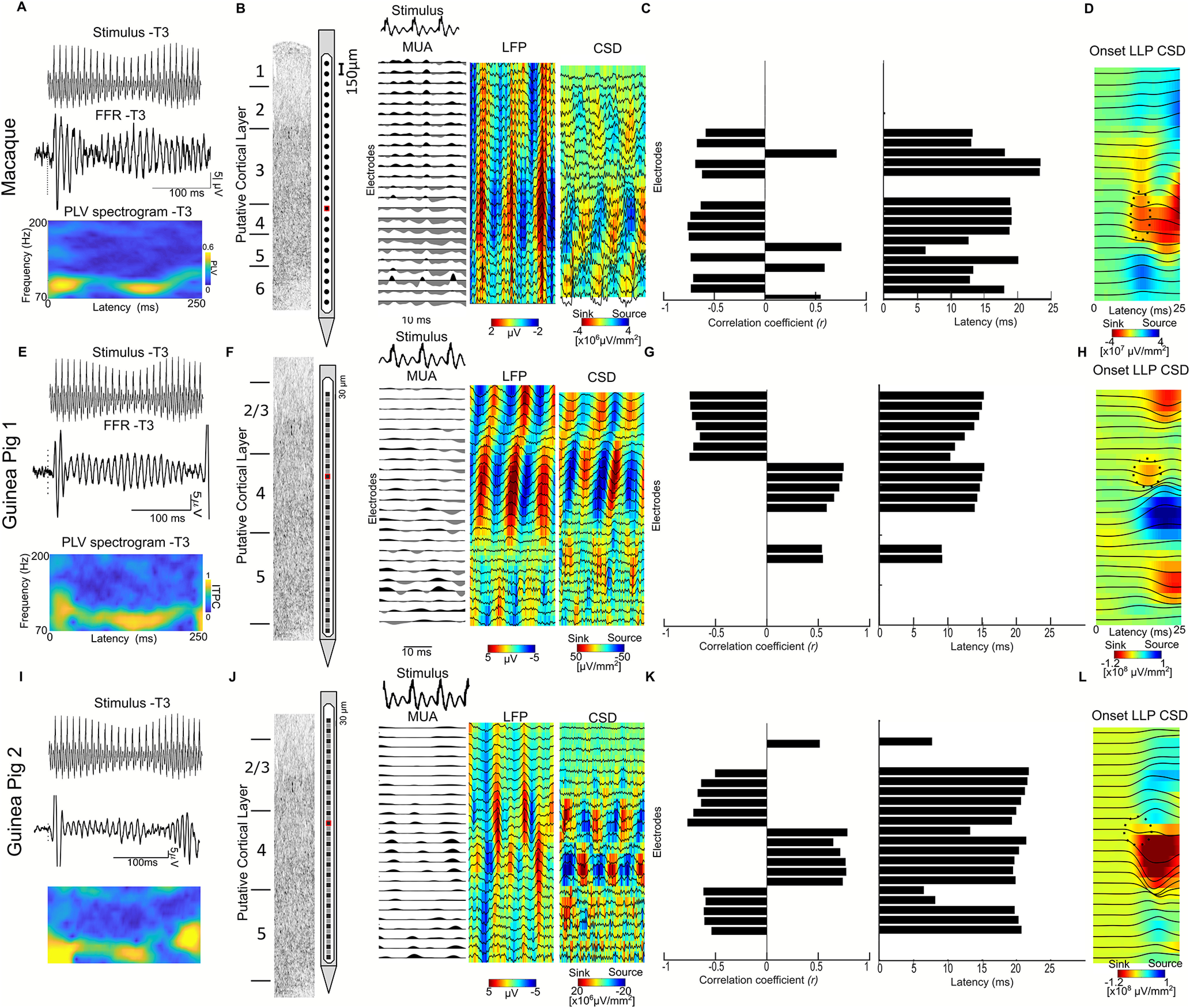
Cortical source for FFRs to speech syllables in the macaque and guinea pig models: FFRs recorded in the macaque and guinea pig models using depth electrodes. ***A***, ***E***, ***I***, Sample stimulus (T3-low-frequency dipping contour) and FFRs with the best signal-to-noise ratios in the putative layer IV in the laminar probe. Waveforms shown are from the electrode with maximum amplitude. Also shown is the inter-trial phase coherence spectrogram for the FFR (electrode shown in red in ***B***, ***F***, and ***J***). ***B***, ***F***, and ***J*** FFRs to stimulus T3, recorded using multichannel laminar-depth electrodes in the macaque and the guinea pig, respectively. The FFRs are shown only for a short segment of the stimulus to clearly visualize the patterns. The MUA and LFPs show strong phase-locked activity to each periodicity cycle in the stimulus. The source-sink configuration in the reference-free CSDs shows the existence of an FFR generator in putative layers III and IV of primary auditory cortex in all three animals. ***C***, ***G***, ***K***, Stimulus to responses correlation coefficients and latencies (lags) between the stimulus and the FFR CSDs in each layer. The shift in the polarity of the CSDs is manifested as a shift in the sign of correlation coefficient and shows sources of FFRs in two cortical layers. ***D***, ***H***, ***L***, CSDs of the long latency potentials (LLPs) to the onset of the Mandarin tones. The first sink (dotted ellipse) in the CSD marks the putative cortical layer IV.

Although intracortical recordings from human subjects provide high spatial and temporal resolution, they are still prone to contamination by volume-conducted fields from the brainstem and subcortical nuclei, and do not provide cortical layer-specific information. To overcome this limitation, we turned to laminar recordings from multicontact electrodes traversing all layers of PAC approximately perpendicular to the cortical sheet (Fig. 4) in the two animal models. These recordings allowed us to compute CSDs, which reflect postsynaptic currents and the corresponding passive return currents in the local cortical populations. Current sinks and sources are independent of volume-conducted potentials from the brainstem and the midbrain. The CSDs can be used to determine whether the postsynaptic currents in cortical populations are phase-locked to the stimulus, and if so, at which cortical depth and latency they arise. In addition, these laminar recordings allowed us to assess the prevalence, latency, and cortical depth of multiunits that phase-lock to the F0 of the stimulus.

Figure 4, *D*, *H*, and *L*, shows CSDs of the low-pass-filtered local field potentials (1–70 Hz) aligned to the stimulus onset. This analysis identified expected patterns of sources and sinks for both animals that were used to identify the putative location of thalamorecipient cortical layers (layer IV and deep layer III), as well as supragranular and infragranular layers. We then computed the CSDs using the same filter setting used for the FFRs. Figure 4, *B* and *J*, shows a 30-ms-long snippet of the CSD FFRs from the sustained portion of tone three for both species (Maq2, GP1, and GP2). Note the presence of several currents that entrained to the F0 of the stimulus (stimulus to CSD correlation, >0.5; Fig. 4*C*,*G*,*K*). We will refer to these currents as cortical frequency-following currents (cFFCs). The most prominent cFFC was located in putative thalamorecipient layers, and a second, somewhat weaker, cFFC with opposite polarity was identified in infragranular layers. There was also an indication of a third and even weaker opposite polarity cFFC in supragranular layers.

The cFFCs in both species showed a strong correlation with the stimulus at latencies of 12–25 ms in the macaque and ∼10–25 ms in the GPs (Fig. 4*C*,*G*,*K*). These latencies are consistent with a cortical origin. Only one infragranular cFFC in macaque had a latency of 6.3 ms that seemed inconsistent with a cortical origin. It is likely that this particular cFFC does not exclusively reflect postsynaptic activity, but rather a very large-amplitude spike that was isolated at this electrode contact and was bleeding into the frequency range of the FFR. Given the short latencies, it is likely that the spike in question corresponded to a passing thalamocortical fiber rather than an infragranular cortical neuron.

In both species, the strongest and most prominent cFFCs were recorded in granular layers, and most likely reflect active postsynaptic currents in response to F0-locked thalamic input at basal dendrites (Fig. 4*B*,*F*,*J*). It is less clear whether the cFFCs in infragranular and supragranular layers reflect active postsynaptic currents, which might be indicative of the propagation of phase-locked responses to these layers, or whether they exclusively reflect passive return currents. In order for the phase-locked activity to spread beyond thalamorecipient layers, not only the postsynaptic input currents but also the output (i.e., their firing rates) would have to be entrained to F0. We thus assessed the frequency following in the MUA, a measure of neural firing rate of small clusters of neurons in the immediate proximity of the electrode contact in the thalamorecipient layers. Electrodes with MUA showing stimulus to response cross-correlation coefficients >0.5 were present in both animal models (*r*_Maq_ = 0.53, Lat_Maq_ = 13.5 ms, *r*_GP1_ = ∼0.53, Lat_GP2_ = ∼13 ms, *r*_GP1_ = ∼0.6, Lat_GP2_ = ∼13 ms). The presence of FFRs in the MUA suggest that the thalamorecipient layers not only receive phase-locked input but also fire in a phase-locked manner to the stimulus F0 in both animal models. These results indicate that the thalamorecipient layers not only receive strong phase-locked input from the thalamocortical fibers but may also propagate the FFRs to downstream cortical layers, albeit with reduced phase-locking strength.

### Relationship between scalp and cortical FFRs

**Figure 5.**
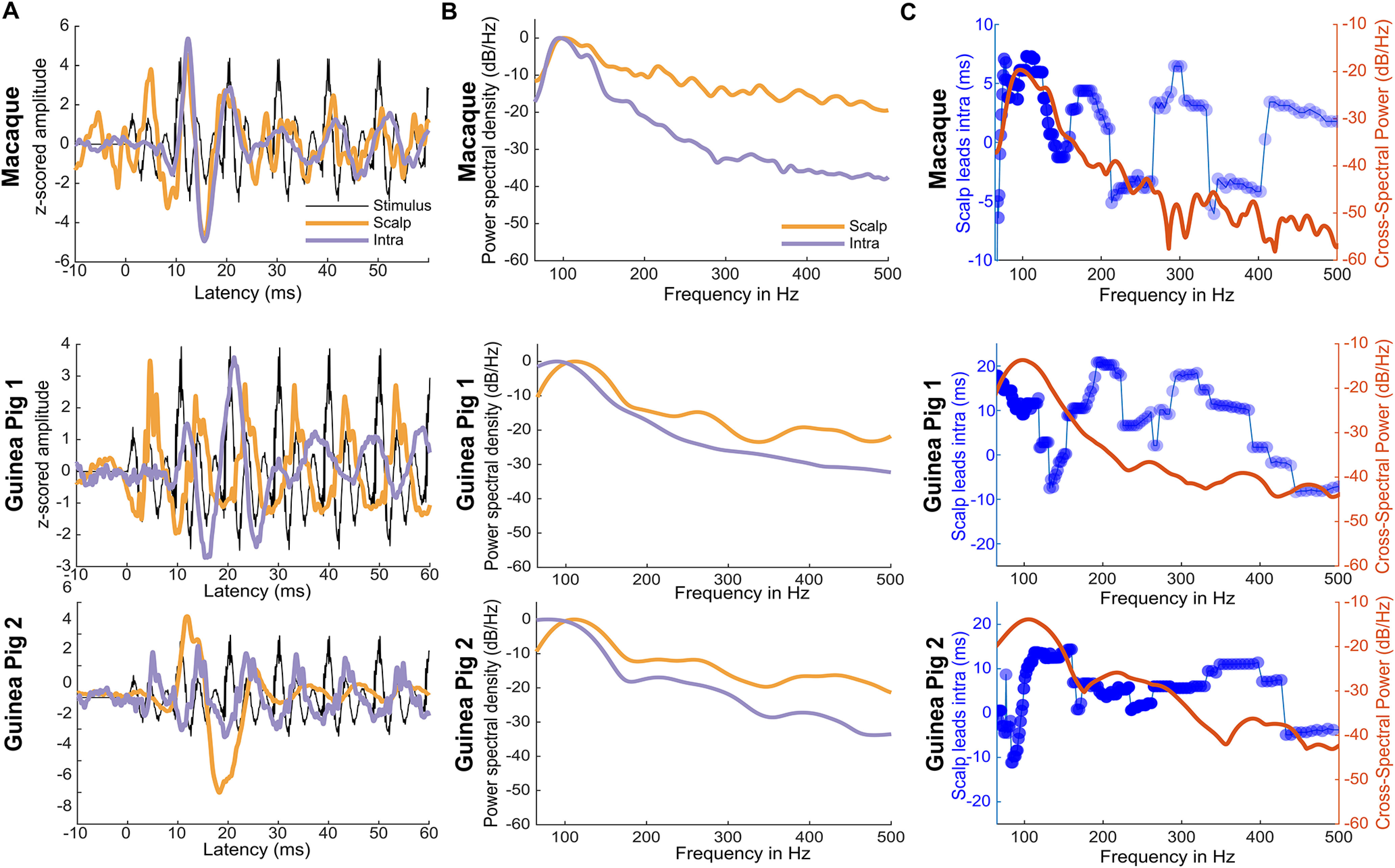
Scalp and intracortical FFRs in the macaque and the guinea pigs. ***A***, FFR waveforms to Mandarin tone 3 (low-dipping F0) at scalp and the cortex in the macaque and the two guinea pigs. ***B***, Normalized power spectral density of the scalp and intracortical FFRs in the macaque and guinea pig, showing the different low-frequency dominance of the intracortical FFRs. ***C***, Difference between scalp-recorded and intracortical FFRs. Blue tracings show the latency between scalp and intracortically recorded FFRs. Circles with darker colors (colors normalized to maximum correlation across frequencies) indicate a higher correlation between the scalp and intracortically recorded FFRs. Intracortical FFRs showed longer latencies than scalp-recorded FFRs at frequencies <120 Hz. Red tracings show the cross-spectral density between scalp and intracortically recorded FFRs showing shared power at low frequencies.

Because both intracranial and scalp FFR recordings were obtained in the same macaque and GPs, we used the opportunity to examine the power and latency across frequencies of the scalp and cortical FFRs to infer similarities. The cortical FFRs were higher in amplitude than the scalp FFRs, presumably because of the proximity of the electrodes to the cortical sources. The comparison of the spectral characteristics of the scalp and cortical FFRs were thus made by normalizing the spectral estimates. Compared with the scalp FFRs, the cFFRs from the PAC were predominantly composed of low-frequency F0 energy relative to higher harmonics (Figs. 1, 5*B*). Figure 5*A* shows the FFRs to tone 3 (low-frequency dipping contour) recorded from the scalp and cortex in the macaque and the GPs. Cortical cFFRs in both species showed longer latencies than the scalp-recorded FFRs (*p* values* *<* *0.01, permutation-based Wilcoxon signed-rank tests on bootstrapped FFR trials; Fig. 5*A*). While the phase-locking of cFFRs to Mandarin tones were higher in the PAC than at the scalp (Fig. 1), the decline in phase-locking with increasing frequency was similar at both the PAC and the scalp. This can also be seen in the difference in normalized power spectral density between the scalp and cortical FFRs at the high frequencies when normalized based on maximum spectral amplitude (Fig. 5*B*).

Cross-spectral power analysis revealed that scalp and cortical FFRs shared strong power coherence with the stimulus near the F0 (70–110 Hz), which declined rapidly at higher frequencies in the cortical FFRs relative to the scalp FFRs (Fig. 5*C*). This trend was similar in all animals (Maq1, GP1, and GP2). This pattern indicated that the FFRs recorded at the scalp and the cortex were similar in power spectral density at the low-frequency regions. Such a pattern can be caused either by a single common source or by more than one source with similar spectral properties but different temporal properties. We thus estimated the cross-correlation strength and latency between the scalp and cortical FFRs across frequencies in the time–frequency domain. The maximum correlation between the scalp and cortical FFRs was seen at frequencies <120 Hz. However, at these frequencies, the cortical FFRs showed delays of ∼7 ms in the macaque and ∼10 ms (Fig. 5*C*) in the GP compared with the scalp FFRs. This indicates the presence of temporally disparate but spectrally overlapping neural sources of the scalp-recorded FFRs. These delays can also be prominently seen in the latencies of the cFFRs, which are higher than conventionally obtained scalp-recorded FFRs (Fig. 5*C*).

**Figure 6.**
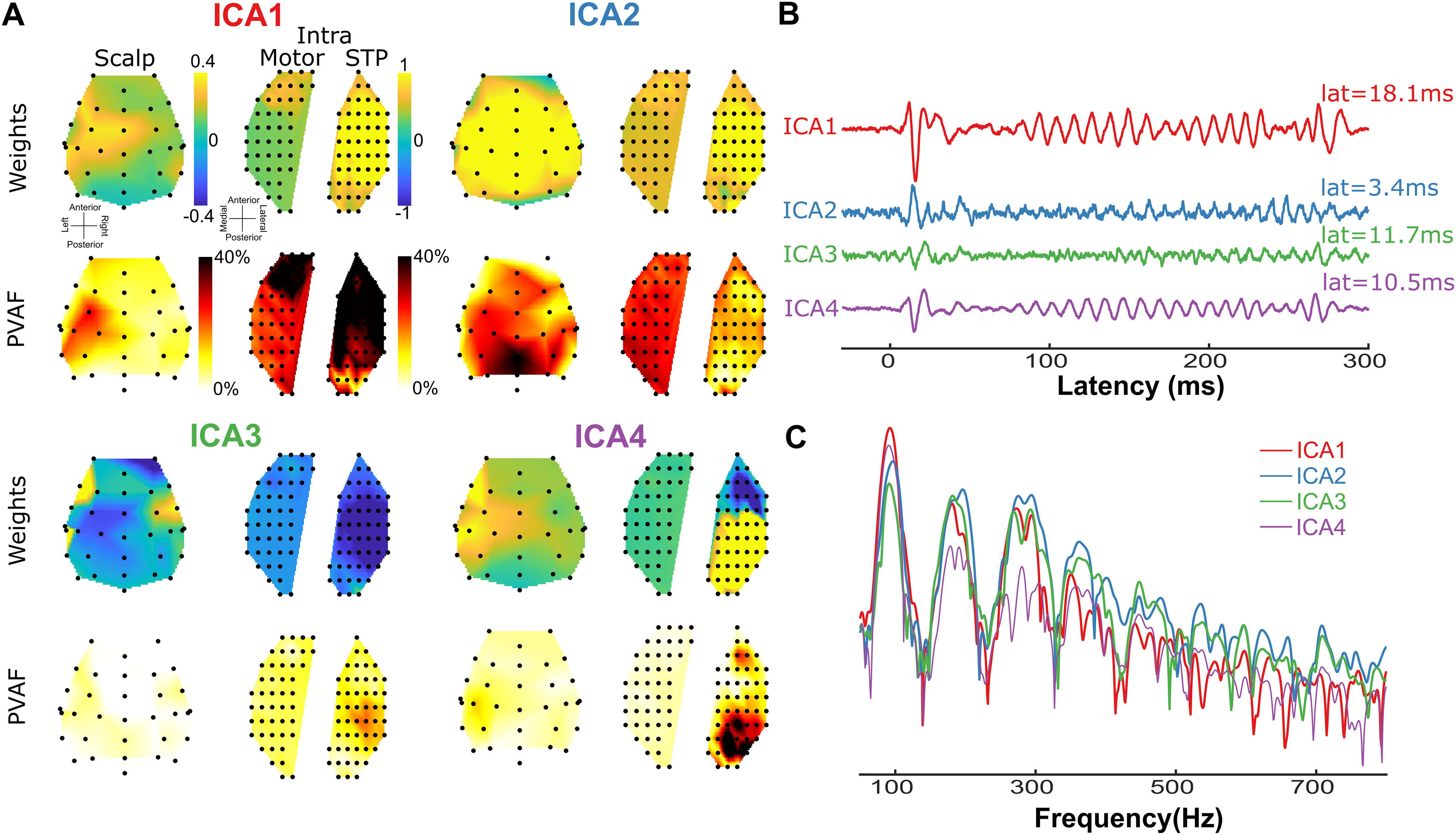
Evaluating the contribution of intracortical FFRs to scalp FFRs using ICA in the macaque. ***A***, Spatial loadings (ICA weights) of the top four independent components onto the scalp and intracortical (intraelectrodes located in the motor regions and in the superior temporal plane) electrodes and the percentage variance accounted for (PVAF) by the ICAs at each electrode. ***B***, Time course of the activations of the top six ICs (amplitude in arbitrary units) for stimulus T3 (low-dipping F0). ***C***, Power spectral coherence of the top six ICAs contributing to the FFRs (amplitude in arbitrary units).

We further applied a blind source separation approach to disentangle the spectrotemporally overlapping components that contributed to the scalp-recorded FFR. We used ICA as the source separation approach (Figs. 6, Maq, 7, GP). In the macaque, we submitted all the scalp (33 electrodes) and intracortical electrodes (96 electrodes spanning the superior temporal plane, and prefrontal and premotor cortex to ICA decomposition. We extracted 12 ICAs that explained 96% of the variance. Among these, we focused on four ICAs, each of which explained >10% of the variance individually and that as a group explained 75% of the variance (Fig. 6). ICA2 was consistent with a volume-conducted generator from the regions distant to all intracranial electrodes (Fig. 6*A*). This is apparent from the widely distributed ICA weights across electrodes. Furthermore, ICA2 had a latency of 3.4 ms Fig. 6*B* and showed prominent power coherence with the stimulus at F0 as well as the higher harmonics (Fig. 6*C*). In contrast, ICA1 had a longer latency (18.1 ms), responded strongest to the F0, and exhibited a steeper gradient between electrodes in the superior temporal plane and motor/premotor cortex. The power coherence with the stimulus also declined in frequency faster than in ICA1. These three findings are consistent with a generator in the primary auditory cortex. This putative cortical ICA1 also propagated to the scalp and contributed to the scalp-recorded FFRs. Similarly, topographies and latencies of ICA4 (Lat = 10.5 ms) suggested a cortical origin and propagated to the scalp electrodes. ICA3 and ICA4, in contrast to ICA1 show spatial weights that are opposite in polarity and hence possibly emerged from different cortical sources with different orientations.

**Figure 7.**
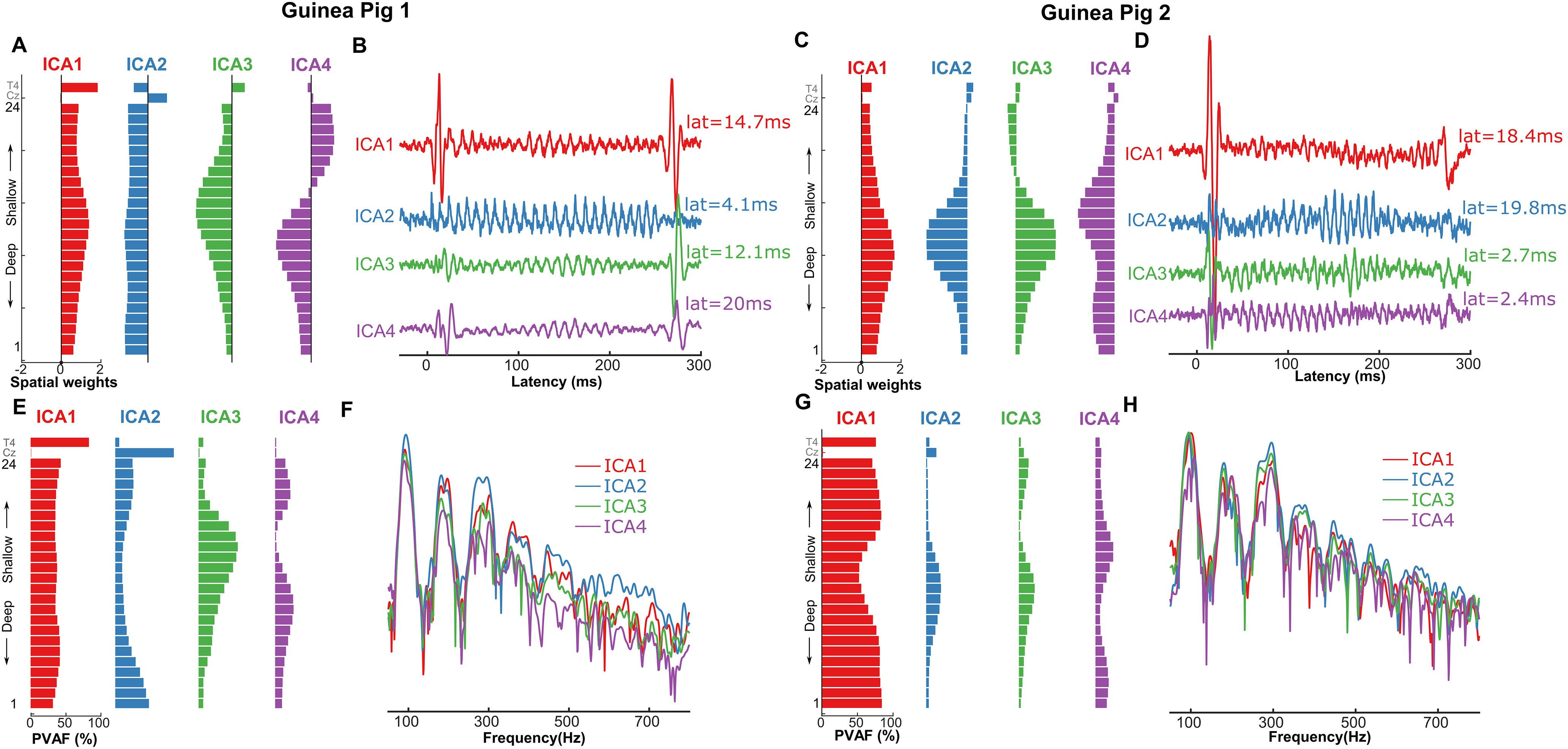
Evaluating the contribution of intracortical FFRs to scalp FFRs using independent component analysis (ICA) in the GP. ***A*** and ***C***, Spatial loadings of the top 4 independent components onto the intracortical laminar electrodes (1–24) and scalp electrodes (Cz and T4). ***B*** and ***D***, Time course of the activations of the top four ICs (amplitude in arbitrary units). ***E*** and ***G***, Percentage variance accounted for (PVAF) of each independent component to the FFRs across the intracortical laminar electrodes. ***F*** and ***H***, Power spectral coherence of the top four ICAs contributing to the FFRs (amplitude in arbitrary units).

In the GP, we submitted the averaged FFRs for each of the Mandarin tones at the 24 electrodes along the layers of the PAC and 2 electrodes placed on the scalp to ICA. The scalp electrodes were placed on the vertex of the scalp (Cz, midpoint of the head along both sagittal and coronal axes) and on the temporal surface of the scalp close to the auditory cortex (T4). Six ICAs were extracted that explained ∼99% of the variance in the FFRs. Among these, the first four components explained >10% of the variance in the FFR amounting to totals of 93.5% (GP1) and 99% (GP2; Fig. 7). Based on visual inspection, the weights of ICA2 in GP1 Fig. 7*A* were not modulated appreciably along the laminar electrode layout. Similarly, ICA3 and ICA4 in GP2 were not modulated appreciably along the laminar electrode layout. This is consistent with volume-conducted activity from distant brain regions, in this case most likely the brainstem (Fig. 7*A*,*C*). It should be noted that the spatial loadings of the volume-conducted ICs in GP2 largely follow the same trend as GP1 but are not exactly similar. This could be in part influenced by the large cortical onset and offset responses that propagate to the scalp in GP2, which are not as apparent in GP1 (Fig. 7*B*, and *D*).

In GP1, the putative subcortical ICA2 had a latency of 4.1 ms and showed strong power coherence with the stimulus F0 as well as the harmonics (Fig. 7*F*). This putative subcortical ICA2 contributed almost the entire variance in the scalp electrodes placed at Cz (Fig. 7*E*), thus suggesting negligent contribution from other sources such as cortex. ICA1 (Lat = 14.7 ms) and ICA3 (Lat = 12.1 ms) showed maximum spatial weights around putative cortical layers 4 and 2/3, and contributed maximally to the variance at the electrode T4 that was placed very close to the surface of primary auditory cortex. In contrast, it did not contribute to variance at the scalp electrode Cz. While ICA4 (Lat = 20 ms) was also consistent with a cortical source, it contributed to very little variance in the scalp electrodes. Together, these results show that in the GP, the scalp electrodes placed at the midline are dominated by the subcortical sources, while those placed on the temporal scalp locations are dominated by cortical sources.

Similar results were also obtained in GP2 where the putative subcortical ICA3 and ICA4 had latencies of 2.7 and 2.4 ms, respectively, and showed strong power coherence with the stimulus F0 as well as the harmonics (Fig. 7*D*). Unlike the volume-conducted ICAs in GP1, the volume-conducted ICAs in GP2 did not explain the entire variance in Cz (Fig. 7*G*). This could be a result of very large onset responses, which skew the ICA decomposition. ICA1 (Lat = 18.4 ms) showed maximum spatial weights around putative cortical layers 4 and 2/3 (Fig. 7*C*), and contributed maximally to the variance at the electrode T4 that was placed very close to the cortical surface of primary auditory cortex. In contrast, it did not contribute to the variance at scalp electrode Cz. While ICA2 (Lat =19.8 ms) was also consistent with a cortical source and contributed to the variance at the scalp (Fig. 7*G* ), it consisted of a late-onset portion and a cortical FFR portion that project to both scalp electrodes. Together, these results show that in the GP animal model, the scalp electrodes placed at the midline are dominated by the subcortical sources, while those placed on the temporal scalp locations are dominated by cortical sources. It should be noted that the SNR of scalp electrodes in GP2 was lower than in GP1 because of the lower number of stimulus sweeps. Also scalp and intracortical FFRs in GP1 were recorded in separate sessions in GP1 while they were recorded simultaneously in GP2. These differences could have driven the subtle differences in ICA patterns between the two GPs.

Thus, the ICA results of both macaques and GPs suggest that the scalp-recorded FFRs contain weighted mixtures of both cortical and subcortical sources. The contributions of both these sources are dependent on the orientation of the net electrical dipole and the location of the scalp electrode. Regardless, the cortical contribution to the FFRs can be seen in both species, and these cortical sources indeed emerge in the auditory cortex and propagate to the scalp.

## Discussion

Research spanning 3 decades has richly characterized the properties of subcortical FFRs (Chandrasekaran and Kraus, 2010; Skoe and Kraus, 2010; Krizman and Kraus, 2019; Coffey et al., 2021), but open questions remain with respect to characteristics of the cortical FFR sources and their contribution to the scalp-recorded FFRs. Our study establishes a cross-species (human, macaque, GP) and cross-level (intracranial, scalp) platform to study cortical FFRs and their contribution to the scalp-recorded FFRs. We present several novel results, as follows: (1) all species readily exhibited FFR-like responses to Mandarin stimuli in both scalp and cortical recordings; (2) better encoding of lower frequencies and longer latencies was a characteristic of the cortical FFRs in all species; (3) the bilateral FFR recordings from the Heschl’s gyrus in humans showed robust encoding and representation of higher fundamental frequencies in the right HG relative to the left HG; (4) RSA revealed striking similarities in the cortical representation of F0 contours, firmly establishing the macaque and guinea pig as viable animal models to study the cortical FFRs to human speech; (5) laminar recordings from the macaque GP auditory cortices demonstrated the existence of cFFCs to human speech sounds in thalamorecipient layers of PAC; and (6) using EEG and large-scale intracranial recordings in the same animal model, we traced the putative contribution of the auditory cortex to the scalp-recorded FFR. Together, our results provide novel insights into the properties of the cortical source of the FFRs to time-varying pitch contours. In the sections below, we highlight and expand on the key findings within and across species.

### Cortical FFRs in humans show distinct low-frequency and right hemisphere bias

Previous studies used distributional source modeling applied to EEG or MEG data to study the cortical source of FFRs (Coffey et al., 2016; Bidelman, 2018; Hartmann and Weisz, 2019). Here, we circumvented the challenges of inverse source localization by using direct intracranial recordings in two human participants and confirmed that robust cortical FFRs to pitch patterns could be evoked in the Heschl’s gyri. These cortical FFRs phase-locked only to the stimulus fundamental frequency, while subcortical FFRs can track speech harmonics as high as 950 Hz (Galbraith et al., 2000; Plyler and Ananthanarayan, 2001). Further, the latencies of cortical FFRs (13–26 ms) were significantly longer than expected of subcortical FFRs (Du et al., 2009; Wang and Li, 2018). Compared with earlier studies, we examined the cortical FFRs to higher F0s (made possible by intracranial recordings) and showed cortical FFRs to F0s as high as 150 Hz.

Bilaterally, the electrodes in the HG showed substantially stronger FFRs compared with those in the PT. No other cortical regions close to the HG showed FFRs. The PT did not phase-lock to the periodicity of the stimulus, which might indicate a transformation of temporal pitch code into a place or a rate-place code in the auditory association cortex. This pattern was also consistent in the macaque data (Maq1), where only electrodes closest to the primary auditory cortex showed strong FFRs. Weaker FFRs on electrodes in motor, premotor, and prefrontal cortex were likely volume-conducted fields not originating in the motor regions.

Consistent with previously reported rightward bias in the cortical FFR activity (Coffey et al., 2016, 2017a,b; Gorina-Careta et al., 2021), we found evidence of distinct rightward asymmetry of FFR magnitudes and steeper phase-locking decline with F0 in the left compared with the right HG. The right hemisphere asymmetry observed in our study may underlie processing differences of melodic and prosodic features in (non-native) speech and music (Zatorre and Belin, 2001; Coffey et al., 2017a).

Experience-dependent changes in FFRs to Mandarin tone stimuli have been extensively used to inform theoretical models of subcortical plasticity (Patel and Iversen, 2007; Krishnan et al., 2012; Skoe and Chandrasekaran, 2014). Our finding of a cortical contribution to FFRs elicited by these very same stimuli adds important new information directly relevant to these theoretical models. As a result, subcortical plasticity models based on the FFRs need to be revisited with a new lens that focuses on the relative cortical and subcortical contributions to experience-dependent plasticity. Given that macaque monkeys can be trained on various auditory tasks and given the similarities of human and monkey FFRs, they are a promising model species to quantify the relative cortical and subcortical contributions to emergent plasticity measured by the FFRs.

It should be noted that earlier intracranial human studies have shown the existence of FFRs to speech at the auditory cortex (Behroozmand et al., 2016; Guo et al., 2021). However, because of the coarse spatial resolution offered by human sEEG, it cannot confirm the presence of cortical frequency following currents as against the thalamocortical input currents, which is essential to firmly establish cortex as a putative generator of scalp-recorded FFRs. By establishing similarities in FFR representation between the human and animal models, and by leveraging high-density laminar recordings in animal models, we were able to explore the laminar sources of the FFRs and break down the cortical contributions to the scalp FFRs with high spatial and temporal detail.

We leveraged RSAs as a translational bridge across levels and species. This allowed us to further deep dive into the FFR sources in animal models at a fine anatomic resolution. Critically, despite differences in recording procedures, anatomy, and arousal states, we demonstrate strong similarities in representational structure between the cortical and scalp FFRs in both human and animal models. Further, the similarity across the species suggests similar representation of the F0 feature in the three species. Further, across the species, the falling tone (T4) was represented less robustly (more confusion) than the rising tone (T2; less confusion), suggesting a cross-species similarity in preferential processing of stimuli with rising, relative to falling pitch (Peng et al., 2018). Because of these similarities, macaques and GPs may be well suited to help answer important questions about the cortical FFRs. The extent of representational similarity across species was lower for scalp-recorded FFRs than intracortically recorded FFRs, which likely reflects a variability in dipole orientations of cortical FFR sources.

These results complement and expand an earlier study (Ayala et al., 2017), which explored the similarity of human and monkey scalp FFRs based on morphologic characteristics of FFR to a single 40 ms/da/syllable with a relatively steady F0. Going beyond a morphologic comparison, we use a range of complex speech sounds with time-varying pitch to assess the species-specific similarity using RSA. We also establish homologies across three animal species along the evolutionary hierarchy, each of which can be leveraged to understand FFRs using advanced approaches that are species specific; for example, optogenetic approaches can be efficiently used in guinea pigs to understand the effects of corticocollicular projections on FFRs, and macaques can be efficiently trained to categorize novel stimuli to reveal the effects of learning on FFRs. We have set a crucial template for future studies to examine the FFRs across species, which is invaluable for leveraging species-specific analytical techniques to comprehensively understand FFR characteristics. Such comprehensive assays of the FFRs are vital to understand the factors underlying altered FFRs in various pathologies and to make the FFRs more readily interpretable and clinically viable.

A caveat regarding the RSA approach is that, unlike the morphologic analysis performed by Ayala et al. (2017), it is agnostic to subtle differences in absolute physical characteristics such as amplitudes, latencies, and phases. This implies that the inference about similarity across species from the RSA alone should not be extrapolated to the specific physical characteristics of the FFRs.

### Cortical FFRs emerge in the thalamorecipient layers of the primary auditory cortex

**Figure 8.**
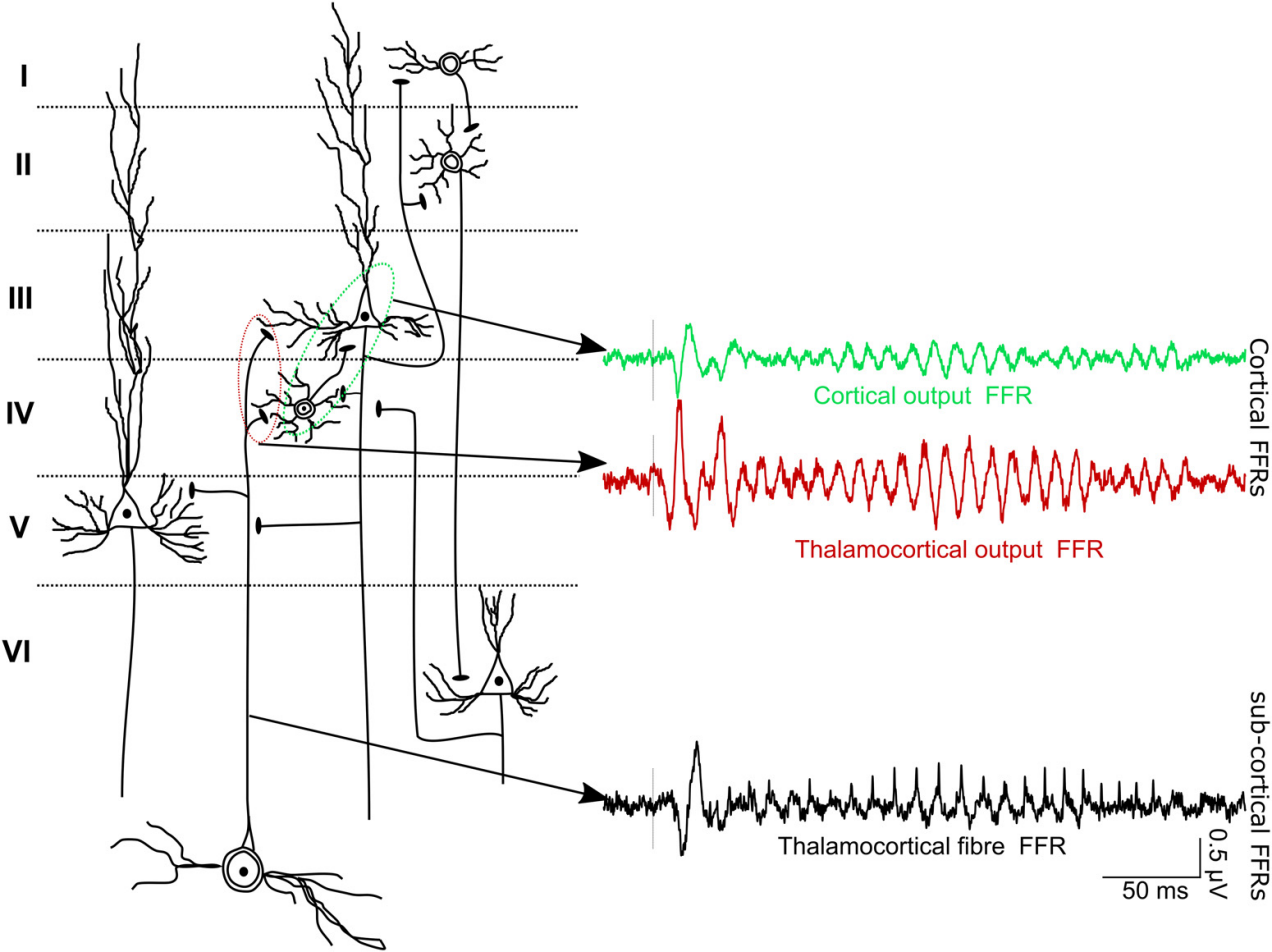
Schematic representation of the cortical FFRs and the subcortical FFRs. Any phase-locked currents in the cortical layers are considered the cortical FFRs. Thalamocortical outputs that synapse with the layers III and IV in the auditory cortex generate postsynaptic potentials that are phase-locked to the stimulus F0.

We consider FFRs as being of cortical origin if they arise from phase-locked postsynaptic currents in cortical neurons, regardless of whether the postsynaptic currents are driven by thalamic or cortical input. Conversely, we define FFRs as having a subcortical origin if they arise from phase-locked postsynaptic currents of neurons located in subcortical nuclei. This working model is schematized in in Figure 8.

With the laminar depth electrodes, we confirmed the existence of cortical FFRs across layers of the PAC. This was consistent in the macaque as well as in both guinea pigs. We found that currents in thalamorecipient layers follow the F0 input from the thalamocortical fibers. The outputs (MUA) of the thalamorecipient layers also follow the stimulus F0. Further, the FFR currents showed phase shifts across the different layers, suggestive of the electric field interferences that may dampen the net equivalent current dipole at the cortex. However, considering the lower strength of the cortical currents beyond the thalamorecipient layer, it is parsimonious to assume that the thalamorecipient layers primarily drive the FFRs recorded at the cortical surface in the human sEEG and macaque. While FFR currents have been demonstrated in earlier work (Steinschneider et al., 1999, 2003) for steady fundamental frequencies <100 Hz, here, we show that cortical FFR currents show phase-locked activity as high as 150 Hz and follow the Mandarin pitch contours. These FFR currents could have partially contributed to the cortical FFR components found in earlier studies using non-invasive assays (Coffey et al., 2016, 2021; Bidelman, 2018; Gorina-Careta et al., 2021) and invasive assays (Behroozmand et al., 2016; Guo et al., 2021). However, it should be noted that the nonlaminar assays (both non-invasive and invasive) could very well pick up both presynaptic and postsynaptic currents that effectively constitute the cortical FFRs (Fig. 8). Nevertheless, we emphasize that the finding of cortical source of the FFR is not at odds with the well established existence of subcortical sources in scalp-recorded FFRs (Marsh et al., 1975; Smith et al., 1978), but show that cortical sources also contribute to scalp FFRs.

### Cortical contributions to extracranially recorded FFRs

The use of animal models enabled us to explore the similarities between the scalp and cortical FFRs in the same subjects. The power coherence between the scalp and cortical FFRs further shows that the cortical sources do not strongly contribute to scalp FFRs at higher harmonics. At the F0, however, there was a strong correlation between the scalp and cortical FFRs. However, the latencies of these correlations suggested that the cortical FFRs had longer latencies than the scalp FFRs.

We used ICA to disentangle the contribution of the spatiotemporally overlapping cortical and subcortical FFR components and their contribution to the scalp-recorded FFRs. We found short-latency volume-conducted ICs that presumably emerged from the subcortical regions and propagated uniformly to all scalp and cortical electrodes. We also found strong ICs that were localized to thalamocortical recipient layers and projected to the scalp. These ICs likely reflect the bulk signal from the cortex that propagates to the scalp. However, not all putative cortical ICs contributed to the scalp-recorded FFRs, because of dipole orientations that did not favor volume conduction to the scalp, and differed based on the electrode location. There was a very specific pattern of differential cortical contribution based on scalp electrode location. In the GP, the midline electrode predominantly picked up the subcortical component, while the electrode above auditory cortex predominantly picked up the cortical component. This effect is driven by the fact that in the GP the auditory cortex is largely on the surface of the temporal lobe with cortical layers oriented mediolaterally unlike humans and other primates where the auditory cortex is buried in the lateral sulcus with cortical layers oriented dorsoventrally (Wallace et al., 2000; Petkov et al., 2006). This further implies that the contribution of the cortical FFRs, though smaller than the subcortical contribution, is present and dependent on the electrode montage used, the location of the auditory cortex and the folding patterns of the cortical surface. The use of two animal models and the multiple modes of intracortical recordings provided complimentary converging evidence for a cortical source of FFRs. At the same time, it sheds light on the species-specific differences and similarities that contribute better understanding of the FFR properties across animal models.

### Limitations and future directions

The sample size can be considered a limitation of the study, potentially limiting the generalizability of the study. However, it is very rare to record FFRs from bilateral Heschl’s gyrus in the same human subject, and we statistically show the comparison between the two hemispheres at a single-subject level. Similarly, we show high-quality replicable recordings in an additional human subject and two macaques and two guinea pigs. Further, we show that the finding of FFRs to speech is localized to the HG similarly in both our human subjects. In addition, the results of both GPs are largely similar. We also report the results of individual humans, macaques, and guinea pigs for better transparency and to avoid overgeneralization across the small sample sizes. We also offer several complementary analyses within and across species to facilitate our analysis, which shows converging evidence for our interpretations. As in all intracranial explorations in human participants, the results are still based on human brains with atypical brains with epileptic seizures, which can hinder generalizability to typical human participants. The macaque and guinea pig models provide an important translation bridge in understanding the laminar profile and the cortical contribution to the scalp FFRs. However, the differences in cortical folding properties and anatomic orientation of the brainstem and the auditory cortices could potentially confound generalization to human participants. We partially circumvent this problem by establishing homologies across the species for both scalp and intracranial FFRs. We did not have simultaneously recorded scalp FFRs in our human sEEG subjects because of clinical challenges and leveraged scalp FFRs from a separate set of participants to understand the differences in scalp and cortical FFRs. Future studies using simultaneously acquired scalp and cortical FFRs will be invaluable in directly inferring about the cortical contribution in human subjects. Further extensive and systematic characterization of the scalp and cortical FFRs would help in better translation of cognitive and behavioral interventions on the neural plasticity at the cortical versus subcortical levels.

### Conclusions

We demonstrate the existence of a cortical source of FFRs using direct intracranial recordings to time-varying pitch patterns. Using an integrated cross-species approach with the macaque and GP models, we show laminar profiles consistent with a cortical source of the FFRs that emerge from the thalamocortical-recipient layers and not from the volume-conducted activity of subcortical neurons. We also show that while subcortical sources dominate the scalp-recorded FFRs, cortical sources do contribute to the scalp-recorded FFRs. Our research paves the way for a wide array of studies to investigate the relevance of this cortical FFR source in auditory perception and plasticity.

## Synthesis

Reviewing Editor: Christine Portfors, Washington State University Decisions are customarily a result of the Reviewing Editor and the peer reviewers coming together and discussing their recommendations until a consensus is reached. When revisions are invited, a fact-based synthesis statement explaining their decision and outlining what is needed to prepare a revision will be listed below. The following reviewer(s) agreed to reveal their identity: NONE. Note: If this manuscript was transferred from JNeurosci and a decision was made to accept the manuscript without peer review, a brief statement to this effect will instead be what is listed below.

The authors have addressed all the previous reviewers' concerns.
